# Thrombus or tumor?

**DOI:** 10.1002/ccr3.7975

**Published:** 2023-09-28

**Authors:** Shiho Wakasa, Hiroya Hayashi, Takanori Yamazaki, Yasuhiro Izumiya, Daiju Fukuda

**Affiliations:** ^1^ Department of Cardiovascular Medicine Osaka Metropolitan University Graduate School of Medicine Osaka Japan

**Keywords:** angioscopy, deep vein thrombosis, pulmonary embolism, tumor thrombus

## Abstract

**Key clinical message:**

Contrast defects in veins are often diagnosed as benign thrombi, but depending on the patient’s background it is necessary to differentiate between tumor thrombi. It is difficult to differentiate between these using contrast‐enhanced CT alone, but with angioscopy it is easy to visually distinguish between a benign and tumor thrombi.

**Abstract:**

Contrast‐enhanced computer tomography (CT) performed on a male patient being treated for de‐differentiated chondrosarcoma revealed contrast defects in the pulmonary artery and right femoral vein, and a diagnosis of pulmonary artery thromboembolism and venous thromboembolism was made, and oral anticoagulant therapy was started. However, a follow‐up CT showed that the contrast defect had extended to the inferior vena cava. Observation using an angioscope revealed that it was not a benign thrombi but a tumor.

## Graphical abstract

A contrast defect was observed in inferior vena cava (IVC) of a 61‐year‐old male patient with de‐differentiated chondrosarcoma, but it was not possible to distinguish whether it was a thrombus or a tumor. Using an angioscope, he was able to clearly diagnose a tumor.

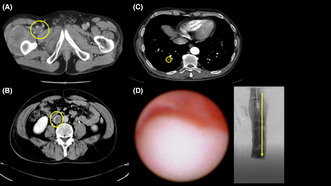

A 61‐year‐old Japanese man visited orthopedics complaining of pain in the right thigh and was diagnosed with dedifferentiated chondrosarcoma (DCS) arising in the right femur after needle biopsy and surgical tumor removal. Preoperative contrast‐enhanced computed tomography (CT) revealed a contrast defect in the right femoral vein (Figure 1A). We suspected that either a benign thrombus or a tumor thrombus had developed involving the inferior vena cava (IVC) and placed a temporary IVC filter (Neuhaus Protect, Toray Medical, Tokyo, Japan) in the perioperative period, and he underwent femoral tumor removal. Twenty days after the operation, contrast‐enhanced CT showed extension of the contrast defect from the right popliteal vein to IVC and new pulmonary embolic findings in the right pulmonary artery (Figure 1B,C). Oral anticoagulant therapy was started, but a follow‐up CT showed no effect. We placed the IVC Filter (Denali, Bard Peripheral Vascular, Inc., Tempe, AZ) and observed the lesion with angioscope (Visible, Intertec Medical, Tokyo, Japan). We diagnosed the patient with a tumor thrombus by angioscopic observation of a smooth‐surfaced, white, elevated lesion in the IVC that had invaded the vein wall (Figure 1D, Video S1). After one course of chemotherapy, the patient refused to continue the treatment and was treated with palliative care. He died of the underlying disease approximately half a year later.

**FIGURE 1:**
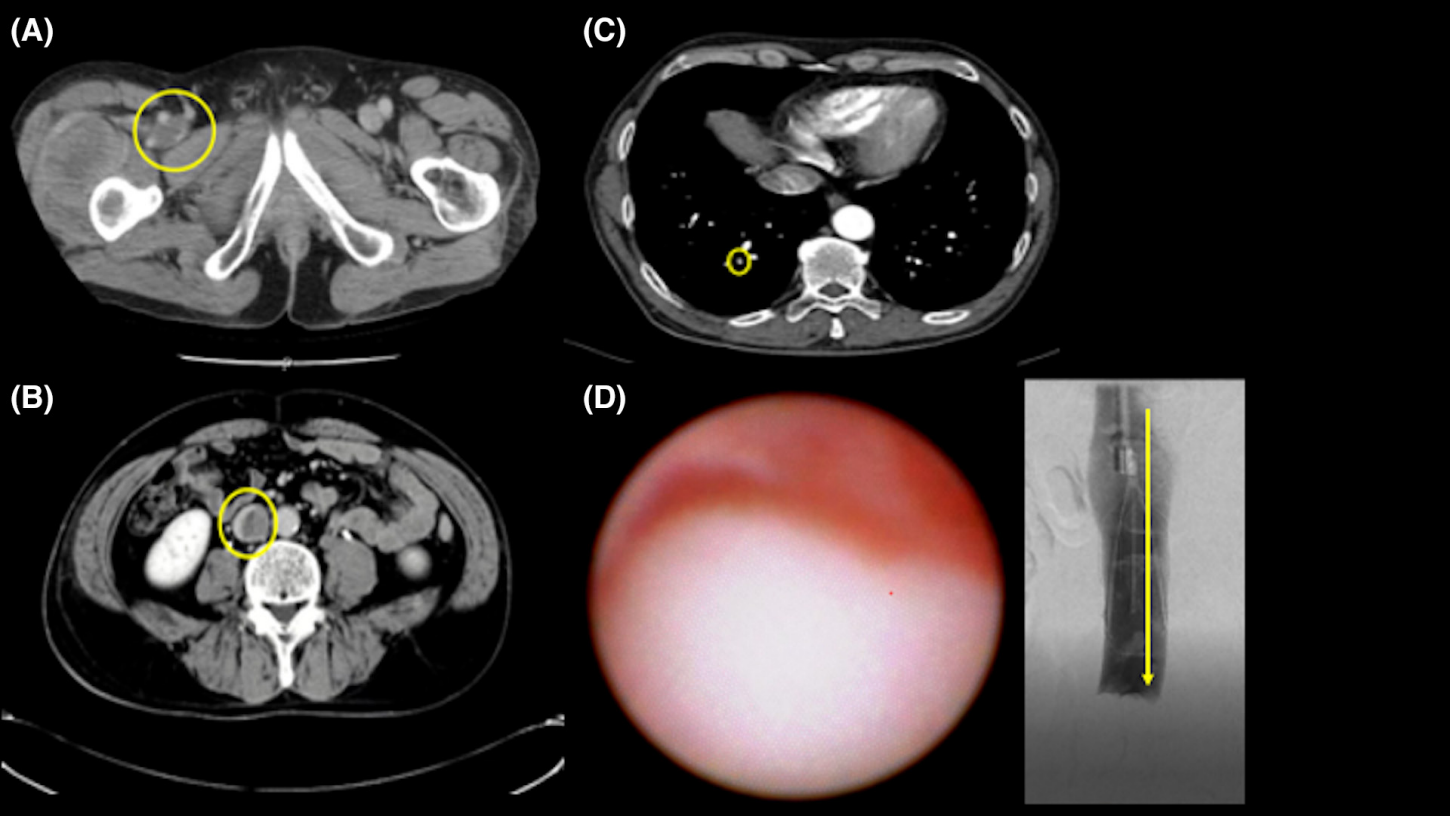
Contrast‐enhanced computed tomography showing images (A–C) and angioscopic image of the tumor thrombus (D). (A) A contrast defect image in the right femoral vein. (B) A contrast defect image in the inferior vena cava. (C) A new pulmonary embolism in right A10. (D) The surface was smooth with white solid components.

Previous reports have demonstrated that FDG‐PET and T2‐weighted MRI can distinguish benign and tumor thrombi.^1^, ^2^ However, clinically, it is not always easy to distinguish between the two. Angioscopy facilitates these distinctions because it visually evaluates. Furthermore, technically, biopsy can be safely performed under angioscopic observation as reported,^3^ although consent was not obtained in this case. Venous thromboembolism is known as a complication secondary to malignant tumors, and anticoagulant therapy is recommended. However, for patients with high bleeding risk or cases in which the diagnosis of the primary disease has not yet been confirmed, angioscope has the potential to serve as a new diagnostic method for tumor thrombus in the future.

## AUTHOR CONTRIBUTIONS

**Shiho Wakasa:** Writing – original draft. **Hiroya Hayashi:** Investigation; methodology; writing – original draft. **Takanori Yamazaki:** Conceptualization; funding acquisition; methodology; project administration; visualization; writing – review and editing. **Yasuhiro Izumiya:** Writing – review and editing. **Daiju Fukuda:** Writing – review and editing.

## FUNDING INFORMATION

This work was supported by a grants‐in‐aid of The Cardiovascular Research Fund, Tokyo, Japan.

## CONFLICT OF INTEREST STATEMENT

The authors declare that they have no conflict of interests.

## ETHICS APPROVAL STATEMENT

This case study was conducted according to the principles of the Declaration of Helsinki and Ethical Guidelines for Medical and Health Research Involving Human Subjects by the Ministry of Health, Labor, and Welfare and Ministry of Education, Culture, Sports, Science, and Technology of Japan. This case study was approved by the Ethics Committee of Osaka Metropolitan University (approval number: 2020–118).

## PATIENT CONSENT STATEMENT

Written informed consent was obtained from the patient to publish this report in accordance with the journal's patient consent policy.

## PERMISSION TO REPRODUCE MATERIAL FROM OTHER SOURCES

Is required for your own works published by other publishers and for which you did not retain copyright.

## CLINICAL TRIAL REGISTRATION

None.

## Supporting information


Video S1.

## Data Availability

The authors confirm that the data supporting the findings of this study are available within the article.
